# Extensive VZV Encephalomyelitis without Rash in an Elderly Man

**DOI:** 10.1155/2014/694750

**Published:** 2014-04-23

**Authors:** Karen Lynch, Prakhar Agarwal, Anu Paranandi, Susan Hadley, Mithila Vullaganti

**Affiliations:** ^1^Department of Neurology, Tufts Medical Center, 800 Washington Street, Boston, MA 02111, USA; ^2^Tufts University School of Medicine, 145 Harrison Avenue, Boston, MA 02111, USA; ^3^Department of Geographic and Infectious Diseases, Tufts Medical Center, 800 Washington Street, Boston, MA 02111, USA

## Abstract

*Introduction.* Varicella zoster virus (VZV) encephalomyelitis with cranial nerve involvement is rare. Characteristically it is preceded by a rash and primarily presents in the immunocompromised. The spectrum of VZV neurologic disease is extensive and it is not uncommon to present without rash. We report the case of an elderly otherwise immunocompetent patient who presented with diverse manifestations of VZV CNS infection all occurring without rash. *Case Report.* A 78-year-old man presented with 1 week of progressive paraparesis and sensory loss, malaise, and fevers. MRI of the neuraxis demonstrated numerous enhancing lesions: intramedullary, leptomeningeal, pachymeningeal, and cranial nerves. Cerebrospinal fluid (CSF) showed a white blood cell count of 420/**μ**L with elevated protein (385 mg/dL). CSF VZV qualitative PCR was positive and CSF VZV immunofluorescence assay detected IgM antibody, confirming the diagnosis of VZV encephalomyelitis. Clinical and radiological improvement was observed after intravenous acyclovir treatment. *Conclusion.* This is a rare report of an immunocompetent patient with extensive VZV encephalomyelitis. We highlight the importance of considering this diagnosis even in the absence of the characteristic rash, and the potential risk of premature discontinuation of antiviral therapy once HSV has been excluded. Prompt recognition and treatment can dramatically reduce morbidity and mortality in patients.

## 1. Introduction

Reactivation of VZV classically presents with herpes zoster (shingles), characterized by pain with an accompanying rash in a dermatomal distribution. However, involvement may extend anywhere along the neuraxis, including the brain (encephalitis, cerebral vasculopathy), cranial nerves (i.e., Ramsay Hunt syndrome, polyneuritis cranialis), cerebellum, spinal cord, and meninges [1]. A rash may or may not be present with VZV reactivation, even with extensive neurologic involvement [2–4].

VZV encephalomyelitis is rare and was traditionally thought to be seen primarily in the immunocompromised patient; however, there have been increasing numbers of this disease entity involving immunocompetent patients as more clinicians utilize CSF PCR and serology to confirm this diagnosis [2]. To date, reports of VZV encephalomyelitis—without an accompanying rash—remain extremely rare; however, with increasing current VZV recognition, the full extent of syndromes of neurologic injury will likely become more evident.

## 2. Case

A 78-year-old man with hypertension and hyperlipidemia presented with a 2-week history of urinary urgency and frequency. Over the next 1 week, he began to experience malaise, headache, otalgia, fever, lower back pain, and bilateral lower extremity weakness with difficulty ambulating and was admitted to his local hospital. He denied sick contacts, rash, or tick bites.

On presentation he had a normal mental status and cranial nerve examination. Pertinent findings noted were diminished sensation to temperature and vibration in the left L5 distribution, impaired proprioception at the toes bilaterally, and areflexia throughout. He had full strength in all extremities. Coordination was intact on finger to nose and heel to shin testing; however, the gait was wide and unsteady.

White blood cell count (WBC) was 12 K/uL. CT and MRI/MRA head were consistent with chronic microangiopathy of the deep white matter and an incidental finding of a right middle cranial fossa arachnoid cyst. CSF profile was noted for a WBC of 115/*μ*L and protein of 633 mg/dL. Glucose and red blood cell counts (RBC) were within normal range. Gram stain and routine bacterial cultures were negative.

One week into his hospital course, his symptoms progressed resulting in right leg plegia, left lower extremity paresis (distal > proximal), and decreased anal tone; his upper extremity strength remained intact. He was subsequently transferred to our main university teaching hospital for further work-up.

Further investigations at our facility comprised of serologies, imaging, CSF, and nerve conduction studies (NCS). Preliminary investigation showed angiotensin converting enzyme and a rheumatologic panel to be negative. Infectious disease work-up testing for Lyme, HIV, chlamydia, gonorrhea, syphilis, and tuberculosis was also negative. Immunologic testing was not performed in this case as the patients' medical history and preliminary blood work did not suggest an immunocompromised state. NCS of the lower extremities revealed a sensorimotor axonal polyneuropathy in the lower extremities.

**Figure 1:**
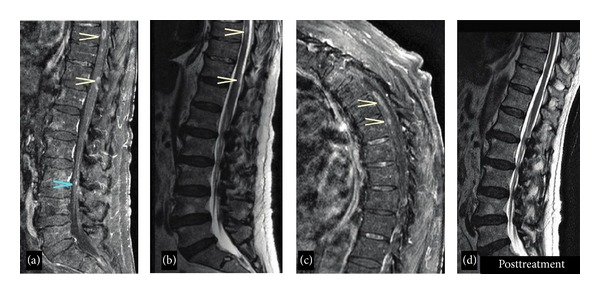
(a) Thoracic and lumbar sagittal MRI and T1 postgadolinium contrast image demonstrating areas of intramedullary enhancement at the levels of T9 and T11, as well as cauda equina enhancement (blue arrow). (b) Sagittal T2 image showing corresponding areas of T2 hyperintensity at the same levels. (c) Sagittal T1 postgadolinium contrast demonstrates enhancement at multiple thoracic levels. (d) Sagittal T2 image after 6 weeks of antiviral treatment demonstrating near resolution of these changes (postcontrast image, not shown, demonstrates resolution of enhancement).

**Figure 2:**
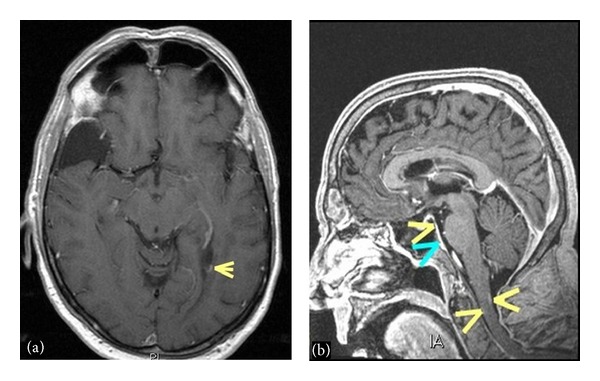
(a) Axial T1 postcontrast image demonstrates an area of ependymal enhancement at the margin of the left lateral ventricle. (b) Sagittal postcontrast T1 volumetric image shows areas of enhancement in the cervical spinal cord (yellow arrows) as well as some areas of pachymeningeal enhancement (blue arrow).

**Figure 3:**
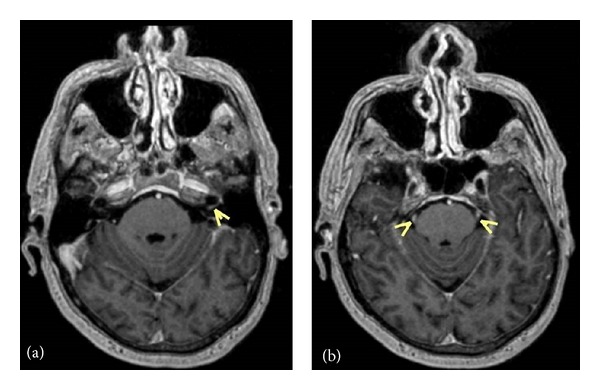
Axial postcontrast T1 volumetric reformatted images demonstrate enhancement of the facial nerve (a) and bilateral trigeminal nerves (b).

MRI of the brain and thoracic and lumbar spine with and without gadolinium was performed. The thoracic and lumbar T1 postcontrast images revealed numerous foci of intramedullary enhancement, including some that were ring enhancing. There was also enhancement of the leptomeninges including the cauda equine (Figure 1). Axial T1 postcontrast brain imaging demonstrated ependymal enhancement at the margin of the left lateral ventricle (temporal horn) as well as enhancement in the cervical spinal cord (yellow arrows) and pachymeningeal enhancement (Figure 2). Additionally, there was abnormal enhancement of the left facial and bilateral trigeminal nerves (Figure 3).

Repeat lumbar puncture (LP) yielded the following results: WBC 420/*μ*L (87% lymphocytes, 5% monocytes, 1% basophils, 0% neutrophils), RBC 10, protein 385 mg/dL, and glucose 47 mg/dL. This was felt to be consistent with an aseptic meningitis, and empiric IV acyclovir 10 mg/kg three times daily was started to treat possible viral etiologies. CSF studies including PCR for Lyme, CMV, EBV, herpes simplex virus (HSV) type 1 (HSV1), HSV 2, human herpes virus 6 (HHV6), and West Nile virus were negative. CSF ACE was within normal limits and CSF VDRL was nonreactive. CSF VZV qualitative PCR was found to be positive and quantitative PCR yielded 1.56 × 10^4^ copies/mL. CSF VZV immunofluorescence assay detected VZV IgM antibody (Quest Diagnostics, Chantilly, VA). A diagnosis of VZV encephalomyelitis was confirmed and IV acyclovir was continued.

During the hospital course the patient showed improvement of the right iliopsoas strength to a medical research council (MRC) grade 3/5 and increased sensation to fine touch in the bilateral lower extremities. However, global areflexia as well as impaired proprioceptive and vibratory sensation in the lower extremities persisted. Additionally, he developed new symptoms of vertical diplopia, and right cranial nerve IV palsy was diagnosed. The patient was discharged to a rehabilitation facility where he completed a 6-week course of IV acyclovir. At his 2-month follow-up appointment, he had significant improvement of his lower extremity strength and was able to ambulate with a walker. Repeat MRI of the thoracic and lumbar spine showed improvement of the intramedullary lesions, both in number and size (Figure 1(c)). The enhancement of the nerve roots of the cauda equina had also decreased. The patient was switched to oral valacyclovir 1 g three times daily for an additional 14 days for extended coverage.

## 3. Discussion

VZV reactivation typically occurs in the immunocompromised host and more commonly this reactivation produces herpes zoster (shingles), resulting in characteristic pain, with or without a rash, in a dermatomal or cranial nerve distribution. While CNS involvement is now considered rare, prior to the introduction of the childhood vaccine in 1995, VZV was reported to be the most common agent associated with acute CNS disease of viral origin—specifically encephalitis, meningitis, and myelitis—followed by herpes simplex virus (HSV), enteroviruses, and influenza A virus [5]. Interestingly, many cases of VZV CNS disease prior to 1995 presented without an accompanying rash [6]. In a recent retrospective study comparing the clinical features of VZV myelitis in immunocompromised versus immunocompetent hosts, the immunocompetent patients were more likely to develop a myelopathy in the absence of rash [7]. With the advent of PCR and immunoassays over the past 15 years, neurologic complications of VZV infection are more frequently recognized. In one series from Finland, VZV was found to be the most common agent identified in viral meningitis and encephalitis (29% of cases) [8], and studies from France and the United Kingdom have implicated VZV in 5–15% of encephalitis [9, 10].

Distinguishing clinically between VZV and HSV encephalomyelitis can be difficult, particularly in the absence of a rash; therefore, certain radiographic findings may be helpful. Radiographic findings of VZV CNS involvement can be extensive, with reports of abnormal T2 hyperintense lesions (with and without gadolinium enhancement) in the brain, spinal cord, and cranial nerves, and diffusion weighted abnormalities due to brain and spinal cord infarctions [6, 11]. VZV, in contrast to HSV, has a predilection for vascular endothelial cells resulting in a cerebral vasculopathy producing multifocal ischemic or hemorrhagic strokes at the grey white junctions. Spinal cord infarction has also been reported as a result of VZV vasculopathy with diffusion weighted abnormalities [12]. HSV infection mainly causes encephalitis and more seldom myelitis. HSV encephalitis tends to involve the orbitofrontal and temporal lobes (necrosis), usually seen as increased T2 signal on MRI with or without gadolinium enhancement, with patients typically presenting with altered mental status and seizures [13]. HSV myelitis secondary to HSV-2 infection is reported as an acute ascending necrotizing pattern of myelitis, with a nonascending transverse myelopathy more commonly found with HSV-1 infection [14].

Virological confirmation is necessary for a diagnosis. Recommended tests included detection of VZV DNA in the CSF (qualitative +/− quantitative PCR), anti-VZV IgM antibody (serum or CSF), and/or anti-VZV IgG antibody in the CSF. Most people have anti-VZV IgG antibody in the serum, and therefore this test is of little clinical value. Detecting anti-VZV IgG antibody in the CSF, however, is a more sensitive indicator of CNS involvement compared to detection of VZV DNA by PCR [12]. One study of 35 CNS VZV cases comparing antibody detection with PCR reported a higher sensitivity of detecting anti-VZV IgG antibody in the CSF (93%) compared with detection of VZV DNA by PCR in the CSF (30%), with a *P* < 0.001 [13]. Given the lower sensitivity of CSF VZV PCR, quantitative viral load estimation by PCR has been proposed as an adjunctive test. Whether a correlation exists between the number of VZV viral copies and severity of neurologic disease is not entirely clear, though it has been proposed [2]. Aberle et al. reported that individuals with >10^4^ CSF viral copies had more severe neurologic manifestations of VZV infection, specifically meningitis and encephalitis [3]. The detection of CSF VZV DNA by PCR, a viral load of 1.5 × 10^4^ copies by quantitative PCR, and detection of anti-VZV IgM antibodies in the CSF confirmed the diagnosis in our patient.

Current guidelines from the Advisory Committee for Immunization Practices (ACIP) recommend a routine single dose of zoster (shingles) vaccine for adults aged 60 years and older, to prevent herpes zoster and postherpetic neuralgia [15]. Incidentally our patient had not received the vaccine. Whether compliance with this guideline will also reduce the incidence of VZV reactivation remains to be seen.

This case emphasizes the fact that VZV reactivation can have extensive CNS involvement in an elderly but otherwise immunocompetent individual, without an accompanying rash. We highlight the importance of considering VZV as a possible cause for encephalomyelitis in the elderly and the recommended laboratory diagnostic testing. A potential pitfall for clinicians is the premature discontinuation of antiviral therapy once HSV has been excluded. Prompt recognition and early treatment can result in significant improvement—both clinically and radiographically—as was observed in our patient, thereby preventing severe or potentially fatal consequences of a disseminated VZV CNS infection.
